# Higher hair cortisol concentrations associated with shorter leukocyte telomere length in high-risk young adults

**DOI:** 10.1038/s41598-022-14905-4

**Published:** 2022-07-11

**Authors:** David Bürgin, Nimmy Varghese, Anne Eckert, Vera Clemens, Eva Unternährer, Cyril Boonmann, Aoife O’Donovan, Marc Schmid

**Affiliations:** 1 Psychiatric University Hospitals, Child and Adolescent Psychiatric Research Department (UPKKJ), University of Basel, Basel, Switzerland; 2 Department for Child and Adolescent Psychiatry/Psychotherapy, University Hospital Ulm, Ulm, Germany; 3 Department of Psychiatry and Weill Institute for Neurosciences, University of California San Francisco, San Francisco, USA; 4 San Francisco Veterans Affairs Medical Center, San Francisco, CA USA; 5 Neurobiological Laboratory for Brain Aging and Mental Health, Transfaculty Research Platform, University of Basel, Basel, Switzerland

**Keywords:** Psychology, Biomarkers, Endocrinology

## Abstract

Chronic stress is associated with accelerated biological aging as indexed by short age-adjusted leukocyte telomere length (LTL). Exploring links of biological stress responses with LTL has proved challenging due to the lack of biological measures of chronic psychological stress. Hair cortisol concentration (HCC) has emerged as a measure of chronic hypothalamic pituitary adrenal (HPA) axis activation, allowing the examination of relationships between aggregate cortisol concentrations over time and LTL. Our sample includes 92 participants (38% women, M_age_ = 26 ± 3.7 years) from a high-risk sample of young adults with previous residential care placements. Two cm hair was collected for HCC, reflecting approximately eight weeks of cortisol secretion. LTL was measured with quantitative polymerase chain reaction (qPCR) in whole blood samples. All samples for LTL were run in triplicate and assayed twice. Linear and polynomial regression models were used to describe the association between HCC and LTL, adjusting for age and sex. HCC and LTL showed negative associations (std. *ß* = − 0.67, 95% CI [− 0.83, − 0.52], p < .001) in age- and sex-adjusted analyses, indicating that higher HCCs are associated with shorter LTL. Using polynomial regression, we found a curvilinear relationship indicating a stronger negative association at lower cortisol concentrations. Higher HCCs were associated with shorter LTL, supporting the hypothesized involvement of prolonged cortisol secretion in telomere attrition. Thus, HCC may prove useful as a biological indicator of chronic stress associated with aging-related processes in samples exposed to high levels of stress.

## Introduction

Chronic stress has been linked with adverse health outcomes, including increased risk for chronic diseases of aging^1–4^. Mounting evidence indicates that accelerated biological aging, as indexed by leukocyte telomere length (LTL), may play a key role in these adverse health effects of stress, however the exact mechanisms remain in part elusive^5–8^. Hypothalamic pituitary adrenal (HPA) axis activation is a putative mechanism conveying stress effects on LTL, since cortisol reactivity to acute stressors is associated with LTL^9^. However, single measures of basal cortisol levels in saliva, blood, and urine samples have not been consistently related to LTL, potentially due to their lack of correspondence with the chronic patterns of HPA axis activation that could accelerate biological aging^9^. In this context, hair cortisol concentration (HCC), as an aggregate measure of stress responding over time, might reveal if chronic HPA axis activation is associated with LTL. To our knowledge, this study is the first study to examine the association between HCC and LTL in humans.

Studies examining associations between psychological stress measures and LTL could improve our understanding of the long-term impact of psychological stress and mental disorders^1,6^. Telomeres are repeated non-coding deoxyribonucleic acid (DNA) sequences that cap the ends of chromosomes and protect the DNA that encodes our genes^10^. Telomeres shorten during cell division, caused by an incomplete replication of the chromosome ends. In this way, telomeres shorten over time, making TL a potential index of cellular age^11,12^. LTL has emerged as an index of cellular age that also has utility as an index of biological age more generally, and of the risk for diseases of aging and mortality^10,13,14^. Short age-adjusted LTL has been linked with exposure to stressors^5,15–18^ and psychiatric disorders^6,8^. Thus, LTL has emerged as an important biomarker of accelerated aging potentially linking psychological stress and psychiatric disorders with increased risk for age-related morbidity.

Decades of research have revealed dysfunction of the HPA axis in numerous mental disorders and following major stressors^19–21^. The HPA axis plays a key role in responses to acute and chronic stressors, resulting in the secretion of the hormone cortisol^19^. Cortisol in turn is involved in homeostasis of various bodily systems and in modulating inflammatory processes and oxidative stress^22,23^. Traditionally, cortisol is measured in saliva, blood, and urine, allowing the assessment of basal levels and reactivity to acute psychological stressors and pharmacological challenges (e.g., dexamethasone)^9^. More recently, hair cortisol concentration (HCC) has emerged as the index of choice for retrospective cortisol accumulation over time, which was previously almost impossible to assess^24–29^.

HPA axis functioning has been implicated in telomere maintenance^30,31^ and there is a burgeoning literature examining associations between cortisol levels and LTL. A recent meta-analysis of 14 studies showed that cortisol reactivity to acute stressors, as measured in saliva, is negatively associated with LTL^9^. However, in the same meta-analysis, no associations between basal cortisol levels in saliva, blood, and urine and LTL were observed. Thus, although differences in acute HPA axis reactivity appear weakly associated with LTL, basal HPA axis activity is not; aggregate measures of cortisol are needed to better understand associations of chronic HPA axis activity with LTL.

To our knowledge, no study has yet examined the association of chronic stress indexed by HCCs with LTL in humans. Moreover, there is a lack of studies investigating the psychophysiological sequelae of stressors and adversities in high-risk populations such as people of low socioeconomic status, racial minorities, and out-of-home placed children and adolescents. This is concerning as these populations are not only underrepresented in research but often overrepresented regarding burden of physical and mental disorders^32–36^. Therefore, this study aims to describe the association between HCCs and LTL in an at-risk sample of young adults with previous child welfare residential placements and high prevalence of mental disorders.

## Methods

### Sample and study procedures

Participants were recruited from a cohort study “Youth Welfare Trajectories: Learning from Experience” that investigates the psychosocial health of young adults with previous residential care placements in Switzerland^37–41^. Blood and hair sampling was conducted between January 2018 and July 2020. A total of 92 participants provided both hair and blood samples for HCCs and LTL assays (35 women [38.0%]; 57 men [62.0%]). The mean age of participants was 26.0 years (SD = 3.7; Range [16.1; 37.6]). The Ethics Commission of Northwestern Switzerland (EKNZ, Ref. 2017-00718) reviewed and approved the study. Written informed consent was provided by all participants.

### Stressor exposures and psychiatric disorders

Childhood and lifetime stressor exposures were measured with the “Childhood Trauma Questionnaire” (CTQ), the “Maltreatment and Abuse Chronology of Exposure Questionnaire” (MACE) and the “Life Events Checklist revised” (LEC-R). Current and past mental disorders were assessed with the “Structured Clinical Interview for DSM5-Disorders- Clinical Version” (SCID5-CV), and the “Structured Clinical Interview for DSM-IV Axis II Disorders” (SCIDII). More detailed information regarding psychosocial measures is found in the Supplementary Material.

### Further descriptives

Smoking behavior was assessed with a short seven item screening questionnaire for nicotine dependence^42^. For descriptive purposes participants were dichotomized into two groups (not/ever smoked) and into current nicotine dependence (yes/no). Somatic complaints were screened with the Massachusetts Youth Screening Instrument-V2 (MAYSI-2) and its six item somatic complaints scale^43^. The scale assesses self-reported and informal bodily aches and pains. Scale specific cut-offs were used to dichotomize participants into non-evident and clinically significant self-reported somatic complaints.

### Hair cortisol concentration

Hair was collected from the posterior vertex region of the scalp. Due to variability on lengths of strands of hair only strands of hair of 2 cm adjacent to the scalp were analyzed reflecting the cortisol secretion over the last eight weeks approximately. Hair cortisol was extracted in line with the protocol by Gao, et al.^44^. In brief, cortisol concertation was determined using a commercially available high-sensitivity (analytical sensitivity 0.007 μg/dL) cortisol enzyme immunoassay kit (Salimetrics Europe, UK) according to the manufacturer’s protocols. Evaporated samples were resuspended in assay diluent provided by the manufacturer. The intra-assay coefficient of variation (CV) was 2.3%, the inter-assay CV was 6.1%. Samples were analyzed in duplicate, and mean values of respective concentrations were calculated in pg/mg hair and used for statistical analyses.

### Leukocyte telomere length measurement

Whole blood was drawn in the morning between 9 and 11 am and subsequently stored at − 80 °C until further use. For the LTL assay, the DNA from whole blood samples was isolated according to the FlexiGene DNA Handbook using the FlexiGene® DNA KIT (250) (Qiagen, DE). LTL was then measured by quantitative polymerase chain reaction (qPCR) according to methods described previously^45–47^. To determine LTL, the T/S ratio (telomere repeat copy number [TELO] to single-copy gene number [SCG; ß-globin]) was calculated for each sample. The exact primer sequences for TELO and SCG are reported in the Supplementary Table S1. The qPCR was performed using the Step One Plus system (Applied Biosystems, USA). All DNA samples were run in triplicate per plate and were assayed twice. The intra-assay CV was < 1%, the inter-assay CV was 4.9%. The threshold cycle (Ct) values were analyzed with the comparative Ct method (2− ∆∆Ct) relative to an internal control to be represented as the T/S ratio. As internal control, a mixture of all DNA samples was used. More detailed descriptions of the DNA extraction and qPCR procedures are found within the Supplementary Material.

### Statistical analyses

**Figure 1:**
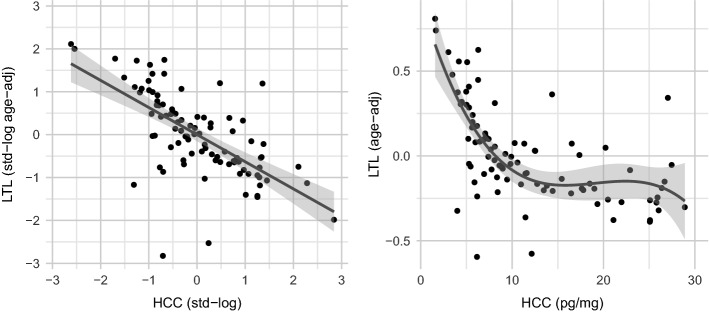
Associations of Hair Cortisol Concentrations (HCCs) and age-adjusted leukocyte telomere length (LTL) in a high-risk sample of young adults: (Left) Scatterplot of log-transformed and standardized age-adjusted LTL and log-transformed and standardized HCC, the regression line corresponds to a linear model displaying the association between HCC and LTL; (Right) Scatterplot of age-adjusted LTL and HCC, the regression line corresponds to a polynomial linear model of third order displaying the association between skewed HCC and LTL.

To describe the association between HCC and LTL we first used correlation analyses, Spearman’s rank correlation (rho) for skewed data and Pearson’s correlation (p) for standardized and log-transformed data. For transformation of skewed biomarkers, we used the natural logarithm to the base e. Descriptives on biomarkers are found in Table 1 and in the Supplementary Material. To test our primary hypothesis that higher HCCs would be associated with LTL, we used a linear regression model to predict LTL by HCC adjusting for age (see Table 2). To describe the observed curvilinear relationship between HCC and LTL of skewed data, a polynomial regression model of third order was fitted to the data (see Table 3). To display these associations, LTL was corrected for age by regressing age on LTL and using the residuals from this model as age-adjusted LTL (see Fig. 1). Post hoc power analyses for these models indicated these models overall and the predictor HCC to be very well powered (see Supplementary Material, page 7 & 8). In secondary analyses, we modelled log-transformed biomarker data including interaction terms and ran a model including sex and the HCC*sex interaction term. In a next model we included age, and an HCC*age-interaction term as further predictors of LTL and then rerun the model stratified by age (cut the 25th and 75th percentile). In further secondary analyses, we investigated if the association between HCC and LTL remained of same magnitude while additionally controlling for diet/nutrition, physical activity, and a socioeconomic status index. The statistical software used was R (Version 4.0.4) through RStudio (Version 1.4.1106, Boston, MA, USA). Correlation analyses and model performance were analyzed using the “easystats” ecosystem for R^48–50^. Plots were created using the “ggplot2” package^52^. P-values for all models are indicated at the levels p < 0.05, p < 0.01, p < 0.001.

**Table 1:** Study descriptives (N = 92).

	%
Sex (women)	38.0%
Childhood Stressors (CTQ)	78.0%
Childhood Stressors (MACE)	90.2%
Current mental disorder (SCID5)	64.1%
Lifetime clinical or personality disorder (SCID5 and SCIDII)	87.0%
Smoking (lifetime)	80.4%
Smoking (current nicotine dependence)	57.6%
Somatic complaints	43.9%
Chronic or acute disease/illness	28.0%
Regular medication	32.5%

	M (SD)
Age	26.0 (3.7)
Potentially traumatic exposures (LEC-R)	4.7 (3.0)
Number of residential care placements	3.7 (3.2)
**Hair Cortisol Concentration (HCC)**^**1**^	13.3 (11.4)
HCC_Women_	15.6 (14.3)
HCC_Men_	11.9 (9.1)
**Leukocyte telomere length (LTL)**^**1**^	0.9 (0.3)
LTL_Women_	0.9 (0.2)
LTL_Men_	0.9 (0.2)

*CTQ* Childhood Trauma Questionnaire, *MACE* Maltreatment and Abuse Chronology of Exposure Scale, *SCID5* Structured Clinical Interview for Mental Disorders based in DSM5, *SCIDII* Structured Clinical Interview for Personality Disorders, *LEC-R* Life-Events Checklist revised. M=Mean; SD=Standard Deviation.

^1^Wilcoxon tests for differences in HCC and LTL between men and women were non-significant.

**Table 2:** Linear regression model predicting standardized z-log LTL by z-log HCC (N = 92).

Predictors	z-log LTL			z-log LTL		
	Std. Beta	CI	p	Std. Beta	CI	p
z-log HCC	− 0.68	− 0.83 to − 0.53	** < 0.001**	− 0.67	− 0.83 to − 0.52	** < 0.001**
Sex [fem.]				0.17	− 0.15 to 0.49	0.282
z-Age				0.11	− 0.05 to 0.27	0.167
R^2^/R^2^ adj	0.462/0.456			0.482 / 0.465		

Significant values are in bold.

*z-log* natural logarithm and standardization, *LTL* leukocyte telomere length, *HCC* hair cortisol concentrations.

**Table 3:** Polynomial regression model predicting LTL by HCC (N = 89).

Predictors	LTL			LTL		
	Beta	CI	p	Beta	CI	p
HCC (1st degr.)	− 0.20	− 0.28 to − 0.12	** < 0.001**	− 0.19	− 0.27 to − 0.11	** < 0.001**
HCC (2nd degr.)	0.01	0.05e^−01^–0.02	** < 0.001**	0.01	0.04e^−01^ to 0.02	**0.001**
HCC (3rd degr.)	− 1.95e^-04^	− 3.27e^−04^ to − 0.61e^-04^	**0.004**	− 1.78e^−04^	− 3.10e^−04^ to − 0.46e^−04^	**0.009**
Sex [fem.]				0.05	− 0.04 to 0.14	0.303
z-Age				0.04	− 0.01 to 0.08	0.106
R^2^/R^2^ adj	0.524 / 0.507			0.546/0.518		

Significant values are in bold.

*z-log* natural logarithm and standardization, *LTL* leukocyte telomere length, *HCC* hair cortisol concentrations.

### Institutional review board

The study was conducted in accordance with the Declaration of Helsinki and approved by the Ethics Committee of Northwestern Switzerland (EKNZ; Ref. 2017-00718; 30.06.2017).

### Informed consent

Informed consent was obtained from all subjects involved in the study.

## Results

### Sample description

Overall, 92 participants provided both hair and blood samples for HCCs and LTL assays (35 women [38.0%]; 57 men [62.0%]). The mean age of participants is in young adulthood (M_age_ = 26.0 years, SD = 3.7; Range [16.1;37.6]). Participants had a mean of 3.7 (SD = 3.2; Range [1;20]) placements in different institutional or foster care settings (see Table 1). Self-reports of child maltreatment showed that 78.0% of participants screened positive for childhood maltreatment using the CTQ, and 90.2% were above the cut-off on at least one of the scales on the MACE. Participants reported a mean of 4.7 potentially traumatic exposures (PTEs) regarding self-experienced or witnessed events on the LEC-R (SD = 2.95; Range [0–12], 5.5% no PTE) (see Table 1). Almost two thirds of the sample was diagnosed with a current DSM5 disorder (64.1%). Lifetime prevalence rates of clinical or personality disorders were high with 87.0% being diagnosed with a current or past disorder. 80.4% of participants reported to smoke, 28% reported to have a chronic or acute illness, 43.9% screened positive for somatic complaints, and 32.5% reported to regularly take some sort of medication. For an overview of study descriptives and data on psychosocial stressors, mental disorders, as well as health-related outcomes and behaviors see Table 1. We did not find significant differences in HCC and LTL scores between the sexes (see Table 1), and in regard to regular medication intake, and current smoking (see Supplementary Material Table S4). We also did not find significant differences between those having a chronic/acute illness and those without, our data however trended towards those self-reporting a chronic disease to show lower HCCs and longer LTL. Taken together, the sample comprises a high-risk sample of young adults with high levels of cumulative stress, and high rates of current and past mental disorders and physical illnesses.

### Associations of higher hair cortisol concentration (HCC) with lower leukocyte telomere length (LTL)

HCC was negatively correlated with LTL. Spearman’s correlation coefficient (rho) of skewed data was − 0.68 (95% Confidence Interval (CI) [− 0.78, − 0.55], p < 0.001). Pearson’s correlation coefficient (r) on log-transformed and standardized HCC and LTL data was − 0.68 (95% CI [− 0.78, − 0.55], p < 0.001). For log-transformed data, a linear model (estimated using observed least square regression [OLS]) was used to predict LTL by HCC first without covariates and in a second step controlling for age and sex (see Table 2). The model including covariates explained a significant and substantial proportion of variance (R^2^ = 0.48, F(3, 88) = 27.34, p < 0.001, adj. R^2^ = 0.46). Within this model, the effect of HCC was significantly negative (*ß* = − 0.67, 95% CI [− 0.83, − 0.52], p < 0.001). To model the relationship of untransformed and skewed HCC and LTL data, a polynomial regression model (estimated using OLS) of third order was fitted to explain the curvilinear relationship observed (see Fig. 1), first without covariates, and in a second step controlling for sex and age (see Table 3). Three data points were omitted in these regressions as their HCC was very high (interquartile range method, HCC > 40 pg/mg). The resulting regression model predicting LTL by HCC, controlling for age and sex, explained a significant and substantial proportion of variance (R^2^ = 0.55, F(5, 83) = 19.94, p < 0.001, adj. R^2^ = 0.52). Modelling these skewed biomarker data, we observed a curvilinear relationship between HCC and LTL, with negative associations at lower HCCs (0–10 pg/mg) and no association at higher HCCs (> 10 pg/mg). The relationship between HCC and LTL in standardized and log-transformed data and using skewed biomarker data is displayed in Fig. 1.

### Secondary analyses

Sex was not found to moderate the association between HCC and LTL in linear models. However, age did significantly moderate the association between HCC and LTL (interaction term: *ß* = − 0.04, 95% CI [− 0.08; − 0.00], p < 0.041). Models in stratified age groups (cut points at the 25th and 75th percentile; age ranges of the three groups: [16.1; 24.9], [25.0; 27.8], [27.9;37.6]) showed a significant negative association between HCC and LTL in participants older than 25 (*ß*_Age [25.0; 27.8]_ = − 0.71, 95% CI [− 0.86, − 0.55], p < 0.001; *ß*_Age [27.9; 37.6]_ = − 1.10, 95% CI [− 1.60, − 0.60], p < 0.001), but not in the youngest age quartile (*ß*_Age [16.1; 24.9]_ = − 0.29, 95% CI [− 0.65, 0.06], p = 0.100). Full model descriptions are provided in Tables S5–S7. In further secondary analyses, we added socioeconomic status, physical activity, and diet/nutrition into the linear regression model (Table 2) to test if controlling for these variables alters the observed association between HCC and LTL. In these models, the effect size for the association between HCC and LTL remained the same (*ß* = − 0.67, 95% CI [− 0.83, − 0.51], p < 0.001), and the explained variance did not markedly increase (see Table S8).

## Discussion

This study aimed to describe the association between HCC and LTL in humans, using data from a high-stress sample of young adults with previous residential care placements. We found a negative relationship between HCC and LTL such that higher HCCs were associated with shorter LTL. Modelling skewed biomarker data, we observed a curvilinear relationship between HCC and LTL, with stronger negative associations between HCCs and LTL at lower HCCs suggestive of a potential threshold effect whereby above a certain level, increasing levels of cortisol are not further associated with telomere shortening in our high-stress sample. Sex did not moderate the association between HCC and LTL; however, the magnitude of the negative association was larger at relatively older ages in our young sample. Results of these secondary analyses must be interpreted with caution. Overall, however, our data are supportive of a relationship between greater chronic HPA axis activity and shorter LTL.

To our knowledge, this is the first demonstration of a relationship between HCCs and LTL. Both elevated cortisol and shorter age-adjusted LTL have been separately linked with early stressors and adversities^5,15,53^, psychiatric disorders^6,8,19^, and chronic diseases of aging in particular^33^. Despite the importance of both the HPA-axis and telomere maintenance for health and their purported associations with one another, studies investigating the interplay of these two systems are inconclusive. Our results extend previous research indicating that cortisol reactivity (in saliva), but not basal cortisol levels, is negatively associated with LTL^9^. Compared with other measures of cortisol (basal levels, cortisol awakening responses, daily cortisol profiles), HCC is emerging as a feasible, non-invasive aggregate measure of chronic patterns of HPA axis activity that can be assessed at a single study visit or remotely^24^. Our findings tentatively support the use of HCC to measure chronic stress associated with aging-related morbidity and underline the potential influence of HPA functioning over time on telomere maintenance.

Considering the strong interest in telomere maintenance and HPA axis functioning as independent outcomes of study, the literature on the interplay of LTL and cortisol is small^9^. The interconnectedness of these two and other systems might help to foster understanding of stress response and resilience trajectories over time^54^. As such for instance, findings of HPA dysregulation as seen in hyper- or hypocortisolism in people exposed to acute versus chronic adversities, stressors, and PTSD, might explain some of the heterogeneity in associations between stressors and telomere maintenance^16,55–57^. In addition to the HPA’s potential direct involvement in telomere maintenance, studies show that the HPA-axis also regulates inflammatory processes and innate immune responses^2,58^. Chronic inflammation, in turn, is a major contributor to the etiology of disease across the life-course^2,59^. In an energetic view of stress, all these systems are embedded and interconnected with mitochondrial functions^60,61^. Future research might benefit from multisystem approaches investigating these different important markers and systems simultaneously over time to better understand dose and time dependence of dysregulation across systems^57,62–64^. Adding to this overall literature, our study adds to our understanding of the interconnectedness of physiological stress systems by investigating two biomarkers of interest, hair cortisol and leukocyte telomere length, in a rare high-risk sample of residential care leavers. This study underlines the need to investigate such intertwined stress processes in high-risk samples and to consider the non-linearity of association in future research.

Our study has some limitations. First, our data are cross-sectional in nature. Directionality of the identified association needs to be examined in studies with longitudinal designs, by investigating trajectories of HCCs and telomere attrition over time. Two studies have shown that higher cortisol reactivity predicts greater telomere shortening over time^65,66^, a finding ripe for replication using HCCs. Second, although post-hoc power analyses revealed the main analyses to be well powered (see Supplementary Material, page 7 & 8, section on post hoc power analysis of regression models”), secondary findings are preliminary in nature and should be interpreted with caution due to the small sample size. Replications of our findings are thus needed to increase validity and generalizability of results. Third, our sample is a highly burdened, high-risk sample of young adults with previous residential and foster care placements and high rates of mental disorders, with no healthy control group available for comparison. Having a healthy control group would strengthen the conclusions of this study. The sample is best described by their overall strain rather than by the presence of any specific stressor or psychiatric diagnosis, this makes it challenging or even impossible to address specificity of effects. However, this sample is well suited to examine how cumulative strains and stressors indexed with an aggregate measure of chronic stress (HCCs), relate to an aggregate measure of accelerated aging as indexed by LTL and thus enhances understanding of the interplay of stress systems and responses in those who have experienced high levels of chronic stress.

To our knowledge, this is the first study to report on an association between HCCs and LTL in humans. Building on previous work on cortisol reactivity and LTL, this study adds to our current understanding of cortisol as a potential contributor to accelerated aging processes as indexed by LTL. Adding HCC as a measure of chronic stress to studies might improve stress phenotyping, especially in high-risk samples. Further clarification of this association in larger, population-based, and longitudinal samples is needed.

## Supplementary Information

Supplementary Information.

## Data Availability

Data are available upon request to the last senior author (MS) using a data sharing agreement specifying the legal bases of data sharing and ensuring the Swiss ethical and data protection standards to be met.
