# Supplementary material for: Higher hair cortisol concentrations associated with shorter leukocyte telomere length in high-risk young adults
Source: Sci Rep. 2022 Jul 11;12:11730. doi: 10.1038/s41598-022-14905-4 (PMC9276815; doi:10.1038/s41598-022-14905-4)
Supplement: Supplementary file 1 — Supplementary Information. [file 41598_2022_14905_MOESM1_ESM.docx]

Hair cortisol and cellular aging – Associations between chronic hypothalamic pituitary adrenal axis activation and telomere length in high-risk young adults

David Bürgin^1,3,4,5^, Nimmy Varghese^2^, Anne Eckert^2^, Vera Clemens^3^, Eva Unternährer^1^, Cyril Boonmann^1^, Aoife O’Donovan^4,5^*, & Marc Schmid^1^*

**Supplementary Methods**

**Psychosocial Measures**

*The Childhood Trauma Questionnaire (CTQ)*

To assess childhood stress exposures, the “Childhood Trauma Questionnaire-Short Form” (CTQ-SF) was used as a retrospective self-report instrument ^1^. The CTQ and its short version CTQ-SF are the most widely used questionnaires internationally to assess experiences of abuse and neglect. Based on the German norm data, severity classifications can be formed ("none-minimal", "slight-moderate", "moderate-severe" to "severe-extreme") ^2^. A cut-off of at least "moderate-severe" was chosen for each of the five subscales, equivalent to other work in large population-based German studies ^2,3^.

*The Maltreatment and Abuse Chronology of Exposure Questionnaire (MACE)*

The MACE is a screening instrument designed to assess interpersonal maltreatment in childhood up to adolescence. It includes 10 subscales: Verbal Violence by Parents, Nonverbal Emotional Violence by Parent, Emotional Neglect, Physical Neglect, Witnessed Physical Violence between Parents, Witnessed Violence to Siblings, Witnessed Violence to Children, Witnessed violence against siblings, Emotional violence by peers, Physical violence by peers, Sexual violence, Loss of a parent. If an experience is specified as having been lived through, the age and the period over which the abuse took place are also asked ^4,5^. Due to the sensitive nature of the questions and their possible stressful effects on the participants' well-being, the questionnaire in was conducted during face-to-face interviews.

*The Life-Events Checklist Revised (LEC-R)*

The LEC-R is a questionnaire that captures the experience of potentially traumatizing events (PTEs) across the life-course ^6^. It contains a list of 19 difficult and stressful events and was implemented as a computer-based screening in the study. All events that were self-experienced or witnessed were summed up to build an overall score of PTEs.

*The Structured Clinical Interview for DSM5-Disorders- Clinical Version (SCID5-CV)*

The SCID-5-CV is a research-constructed, semi-structured clinical interview based on the adult DSM-5 disorders ^7^. It captures the following 10 dimensions: Affective Episodes, Psychotic and Associated Symptoms, Differential Diagnosis of Psychotic Disorders, Differential Diagnosis of Affective Disorders, Disorders Associated with Psychotropic Substances, Anxiety Disorders, Obsessive-Compulsive Disorder and Post-Traumatic Stress Disorder, Attention-Deficit/Hyperactivity Disorder, Screening for Other Disorders, Adjustment Disorder.

*The Structured Clinical Interview for DSM-IV Axis II Disorders (SCID-II)*

The semi-structured interview SCID-II was used to assess Personality Disorders ^8^. The interview consists of 134 questions, which are rated on a four-point scale (0 = not assessable, 1 = criterion not met, 2 = criterion partially met, 3 = criterion met). If the number of items coded as 3 exceeds the threshold provided in DSM-IV, the diagnosis of the corresponding PS is assigned.

**Detailed Telomere Length Measurement Procedure**

*DNA Isolation*

The DNA of the cells was isolated according to the FlexiGene DNA Handbook using the FlexiGene^®^ DNA KIT (250) (Qiagen, DE). Briefly, 300 ul of whole blood was mixed into 750 μl buffer FG1 and was centrifuged at 10000 *x* g for 20 s. In the next step, 150 μl FG2/GIAGEN protease was mixed into the tube and then incubated at 65°C for 5 min. Afterwards, 150 μl of isopropanol (100 %) was added. Then, the tube was vortexed until a DNA precipitate was observed. This step was followed by a centrifugation for 5 min at 10000 *x* g. The supernatant was discarded, and the pellet was resuspended in 150 μl of 70 % ethanol by vertexing for 5 sec. After discarding the supernatant, the DNA pellet was air-dried until complete liquid evaporation. The DNA pellet was then dissolved in 200 μl buffer FG3 by vertexing for 5 sec at low speed, followed by an incubation for 60 min at 65 °C in a heating block. The DNA content was assessed by using a Nanodrop 1000 spectrophotometer (Thermo Fisher Scientific, USA). All DNA samples were stored in -80°C until further procedure.

*Leukocyte Telomere Length*

Telomere length was measured by quantitative polymerase chain reaction (qPCR) according to the method described previously ^9-11^. To determine telomere length, the T/S ratio (telomere repeat copy number (TELO) to single-copy gene number (SCG)) was assessed. The TELO and SCG was detected for each DNA sample. Table 1 lists the forward and reverse primers of TELO and SCG (ß-globin). For the qPCR, a master mix was prepared. The composition of one reaction of TELO master mix was 1.08 ul teloF primer (54 nM), 1,6 μl teloR primer (80 nM), 5,32 μl H2O and 10 ul SYBR green master mix and that of the SCG master mix was 1,6 μl ß-globinF primer (80 nM), 1,6 μl ß-globinR primer (80 nM), 4,8 μl H2O and 10 μl SYBR green master mix. The master mixes of TELO or SCG (18 μl per well) were loaded into 96-well-plates. Afterwards, 2 μl of the DNA sample, at a final concentration of 10 ng/μl, was added to the corresponding wells. The qPCR was performed using the Step One Plus system (Applied Biosystems, USA). All DNA samples were run in triplicate and were assayed twice at two different time points. The qPCR settings were first the initial denaturation at 95°C for 10 min, followed by a cycling of 50 repeats containing 1.) 10 sec hold at 95°C and 2.) 60 sec hold at 58 °C. The Ct values were exported with the SABiosciences PCR Array Data Analysis Software and analyzed with the comparative Ct method (2^- ∆∆Ct^) relative to an internal control to be represented as the T/S ratio. As internal control, a mixture of all DNA samples was used.

**Table S1.** Forward and reverse primer sequences of TELO and SCG.

| **Primer** | **Sequence (5’-3’)** |
| --- | --- |
| teloF | CGGTTTGTTTGGGTTTGGGTTTGGGTTTGGGTTTGGGTT |
| teloR | GGCTTGCCTTACCCTTACCCTTACCCTTACCCTTACCCT |
| ß-globinF | GCTTCTGACACAACTGTGTTCACTAGC |
| ß-globinR | CACCAACTTCATCCACGTTCACC |

**Table S2.** Materials used in DNA isolation and qPCR procedure

| Material | Company | Lot |
| --- | --- | --- |
| FlexiGene^®^ DNA KIT (250) | Qiagen, DE | 163051128 |
| Nanodrop 1000 spectrophotometer | Thermo Fisher Scientific, USA | - |
| Primers | Microsynth AG, CH | 3301770,3301772,3241899,3241900 |
| SYBR green master mix | Applied Biosystems, USA | 2010603 & 2010604 |

**Supplementary Results**

**Table S3.** Detailed descriptives on HCC and LTL.

|  | **HCC** | **zlog-HCC** | **LTL** | **z-log LTL** |
| --- | --- | --- | --- | --- |
| Mean | 13.3 | 0 | 0.89 | 0 |
| SD | 11.4 | 1 | 0.3 | 1 |
| Median | 9.36 | -0.12 | 0.82 | -0.08 |
| Min | 1.57 | -2.61 | 0.36 | -2.63 |
| Max | 78.21 | 2.84 | 1.73 | 2.19 |
| Range | 76.64 | 5.46 | 1.37 | 4.82 |
| Skew | 2.76 | 0.16 | 0.76 | 0.08 |
| Kurtosis | 11.02 | 0.04 | -0.06 | -0.45 |

**Notes.** z-log = natural logarithm and standardization; LTL = leukocyte telomere length;

HCC = hair cortisol concentrations.; SD = Standard Deviation.

**Table S4.** Detailed descriptives on HCC and LTL by select study

descriptives.

|  | **HCC**  M (SD) | **LTL**  M (SD) |
| --- | --- | --- |
| Chronic/Acute Illness/Disease |  |  |
| Yes | 9.47 (5.68) | 0.96 (0.32) |
| No | 15.01 (12.85) | 0.86 (0.29) |
| *Wilcoxon Test* | p = .067 | p = .066 |
| Regular Medication |  |  |
| Yes | 12.5 (9.45) | 0.9 (0.32) |
| No | 13.92 (12.48) | 0.86 (0.27) |
| *Wilcoxon Test* | p = .783 | p = .865 |
| Smoking (Current) |  |  |
| Yes | 13.4 (12.07) | 0.89 (0.29) |
| No | 13.05 (10.73) | 0.89 (0.31) |
| *Wilcoxon Test* | p = .939 | p = .888 |

**Notes.** z-log = natural logarithm and standardization; LTL = leukocyte telomere

length; HCC = hair cortisol concentrations; SD = Standard Deviation.

**Table S5.** Linear Regression Model predicting standardized (z)-log

LTL by z-log HCC, Sex, and an HCC*Sex Interaction (N=92).

|  | **z-log LTL** | | |
| --- | --- | --- | --- |
| *Predictors* | *Std. Beta* | *CI* | *p* |
| z-log HCC | -0.71 | -0.91 – -0.51 | **<0.001** |
| Sex [female] | 0.20 | -0.12 – 0.53 | 0.219 |
| z-log HCC * Sex [female] | -0.01 | -0.35 – 0.32 | 0.942 |
| Observations | 92 | | |
| R^2^ / R^2^ adjusted | 0.471 / 0.453 | | |

**Notes.** z-log = natural logarithm and standardization; LTL = leukocyte

telomere length; HCC = hair cortisol concentrations.

**Table S6.** Age Moderations in the association of HCC and LTL.

|  | **z-log LTL** | | |
| --- | --- | --- | --- |
| *Predictors* | *Std. Beta* | *CI* | *p* |
| z-log HCC | 0.44 | -0.64 – 1.53 | 0.416 |
| Age | 0.03 | -0.01 – 0.07 | 0.182 |
| z-log HCC * Age | -0.04 | -0.08 – -0.00 | **0.041** |
| Observations | 92 | | |
| R^2^ / R^2^ adjusted | 0.500 / 0.483 | | |

**Notes.** z-log = natural logarithm and standardization; LTL = leukocyte

telomere length; HCC = hair cortisol concentrations.

**Table S7.** Association of HCC and LTL stratified by Age.

|  | **z-log LTL** | | |
| --- | --- | --- | --- |
| *Predictors* | *Std. Beta* | *CI* | *p* |
| z-log HCC [under 25^th^ age percentile] (N=23) | -0.30 | -0.66 – 0.06 | 0.100 |
| z-log HCC [25^th^ to 75^th^ age percentile] (N=46) | -0.72 | -0.88 – -0.57 | **<0.001** |
| z-log HCC [above 75^th^ age percentile] (N=23) | -1.12 | -1.63 – -0.61 | **<0.001** |

**Notes.** z-log = natural logarithm and standardization; LTL = leukocyte telomere length; HCC = hair

cortisol concentrations.

**Table S8.** Association of HCC and LTL controlled for age, sex, physical

activity, diet, and socioeconomic status.

|  | **z-log LTL** | | |
| --- | --- | --- | --- |
| *Predictors* | *Std. Beta* | *CI* | *p* |
| z-log HCC | -0.67 | -0.83 – -0.51 | **<0.001** |
| Sex [fem.] | 0.25 | -0.08 – 0.59 | 0.140 |
| Age | 0.04 | -0.14 – 0.22 | 0.655 |
| Diet/Nutrition * | -0.17 | -0.33 – -0.01 | **0.042** |
| Physical Activity ** | 0.02 | -0.15 – 0.18 | 0.823 |
| SES index *** | 0.17 | -0.02 – 0.35 | 0.078 |
| Observations | 85 | | |
| R^2^ / R^2^ adjusted | 0.514 / 0.476 | | |

**Notes.** z-log = natural logarithm and standardization; LTL = leukocyte telomere length;

HCC = hair cortisol concentrations; SES = socio-economic status. ^*^ Diet/Nutrition was

assessed with a short screener from Blackburn and Epel ^12^, higher scores relate to better

nutrition; ^**^ Physical Activity was measured with the Stanford leisure-time activity categorical

item ^13^ higher scores indicate more activity; *** SES was measured with a single item in the

anamnesis on a categorical item relating to their monthly income aligned with Swiss standards,

higher scores indicate a higher SES.

**Post hoc Power Analysis of Regression Models**

**F tests** - Linear regression (Table 2; uncontrolled): Fixed model, R² deviation from zero

**Analysis:** Post hoc: Compute achieved power

**Input:** Effect size f² = 0.8587361

α err prob = 0.05

Total sample size = 92

Number of predictors = 1

**Output:** Noncentrality parameter λ = 79.0037212

Critical F = 3.9468757

Numerator df = 1

Denominator df = 90

Power (1-β err prob.) = 1.0000000

**t tests** - Linear bivariate regression (Table 2; uncontrolled): One group, size of slope

**Analysis:** Post hoc: Compute achieved power

**Input:** Tails = Two

Slope H1 = -0.68

α err prob = 0.05

Total sample size = 92

Slope H0 = 0

Std dev σ_x = 1

Std dev σ_y = 1

**Output:** Noncentrality parameter δ = -8.8955580

Critical t = -1.9866745

Df = 90

Power (1-β err prob.) = 1.0000000

**F tests** - Linear multiple regression (Table 2; controlled for age and sex): Fixed model, R² deviation from zero

**Analysis:** Post hoc: Compute achieved power

**Input:** Effect size f² = 0.9305019

α err prob = 0.05

Total sample size = 92

Number of predictors = 3

**Output:** Noncentrality parameter λ = 85.6061748

Critical F = 2.7081865

Numerator df = 3

Denominator df = 88

Power (1-β err prob) = 1.0000000

**t tests** - Linear multiple regression (Table 2; controlled for age and sex): Fixed model, single regression coefficient

**Analysis:** Post hoc: Compute achieved power

**Input:** Tail(s) = Two

Effect size f² = 0.8920686

α err prob = 0.05

Total sample size = 92

Number of predictors = 3

**Output:** Noncentrality parameter δ = 9.0592666

Critical t = 1.9872899

Df = 88

Power (1-β err prob) = 1.0000000

**F tests** - Linear multiple regression (Table 3, uncontrolled): Fixed model, R² deviation from zero

**Analysis:** Post hoc: Compute achieved power

**Input:** Effect size f² = 1.10084

α err prob = 0.05

Total sample size = 89

Number of predictors = 3

**Output:** Noncentrality parameter λ = 97.9747600

Critical F = 2.7119214

Numerator df = 3

Denominator df = 85

Power (1-β err prob) = 1.0000000

**F tests** - Linear multiple regression (Table 3; controlled for age and sex): Fixed model, R² deviation from zero

**Analysis:** Post hoc: Compute achieved power

**Input:** Effect size f² = 1.202643

α err prob = 0.05

Total sample size = 89

Number of predictors = 5

**Output:** Noncentrality parameter λ = 107.0352

Critical F = 2.3244732

Numerator df = 5

Denominator df = 83

Power (1-β err prob) = 1.0000000

**References**

1 Bader, K., Hänny, C., Schäfer, V., Neuckel, A. & Kuhl, C. Childhood trauma questionnaire–psychometrische Eigenschaften einer deutschsprachigen Version. *Zeitschrift für Klinische Psychologie und Psychotherapie* **38**, 223-230 (2009).

2 Hauser, W., Schmutzer, G., Brahler, E. & Glaesmer, H. Maltreatment in childhood and adolescence: results from a survey of a representative sample of the German population. *Dtsch Arztebl Int* **108**, 287-294, doi:10.3238/arztebl.2011.0287 (2011).

3 Witt, A., Brown, R. C., Plener, P. L., Brahler, E. & Fegert, J. M. Child maltreatment in Germany: prevalence rates in the general population. *Child Adolesc Psychiatry Ment Health* **11**, 47, doi:10.1186/s13034-017-0185-0 (2017).

4 Isele, D. *et al.* KERF–ein Instrument zur umfassenden Ermittlung belastender Kindheitserfahrungen. *Zeitschrift für Klinische Psychologie und Psychotherapie* (2014).

5 Teicher, M. H. & Parigger, A. The 'Maltreatment and Abuse Chronology of Exposure' (MACE) scale for the retrospective assessment of abuse and neglect during development. *PLoS One* **10**, e0117423, doi:10.1371/journal.pone.0117423 (2015).

6 Gray, M. J., Litz, B. T., Hsu, J. L. & Lombardo, T. W. Psychometric properties of the life events checklist. *Assessment* **11**, 330-341, doi:10.1177/1073191104269954 (2004).

7 First, M., Williams, J., Karg, R. & Spitzer, R. Structured clinical interview for DSM-5 disorders, clinician version (SCID-5-CV). *Arlington, VA: American Psychiatric Association* (2015).

8 Wittchen, H., M, Z. & T, F. Strukturiertes Klinisches Interview fu ̈r DSM-IV (SKID-I und SKID-II). *Göttingen: Hogrefe* (1997).

9 Axelrad, M. D., Budagov, T. & Atzmon, G. Telomere length and telomerase activity; a Yin and Yang of cell senescence. *J Vis Exp*, e50246, doi:10.3791/50246 (2013).

10 O'Callaghan, N. J. & Fenech, M. A quantitative PCR method for measuring absolute telomere length. *Biol Proced Online* **13**, 3, doi:10.1186/1480-9222-13-3 (2011).

11 Cawthon, R. M. Telomere measurement by quantitative PCR. *Nucleic Acids Res* **30**, e47, doi:10.1093/nar/30.10.e47 (2002).

12 Blackburn, E. & Epel, E. *The telomere effect: a revolutionary approach to living younger, healthier, longer*. (Hachette UK, 2017).

13 Kiernan, M. *et al.* The Stanford Leisure-Time Activity Categorical Item (L-Cat): a single categorical item sensitive to physical activity changes in overweight/obese women. *International Journal of Obesity* **37**, 1597-1602 (2013).
